# Occupational Hazards and Risks Associated with Phthalates among Slovakian Firefighters

**DOI:** 10.3390/ijerph17072483

**Published:** 2020-04-05

**Authors:** Branislav Kolena, Ida Petrovičová, Miroslava Šidlovská, Henrieta Hlisníková, Lenka Bystričanová, Soňa Wimmerová, Tomáš Trnovec

**Affiliations:** 1 Department of Zoology and Anthropology, Constantine the Philosopher University in Nitra, 949 74 Nitra, Slovakia; ipetrovicova@ukf.sk (I.P.); msidlovska@ukf.sk (M.Š.); henrieta.hlisnikova@ukf.sk (H.H.); lenka.bystricanova@student.ukf.sk (L.B.); 2 Institute of Biophysics, Informatics and Biostatistics, Slovak Medical University in Bratislava, 833 03 Bratislava, Slovakia; sona.wimmerova@szu.sk; 3 Department of Environmental Medicine, Slovak Medical University, 833 03 Bratislava, Slovakia; tomas.trnovec@szu.sk

**Keywords:** phthalate exposure, firefighters, biomonitoring, health, occupational

## Abstract

Despite increasing attention to the occupational risk of firefighters, little is known about phthalate exposure. In our study, we detected mono-isobutyl phthalate (MiBP), mono-n-buthyl phthalate (MnBP), mono(2-ethyl-5-hydroxyhexyl) phthalate (5OH-MEHP), mono(2-ethyl-5-carboxypentyl) phthalate (5cx-MECPP), and mono(2-ethyl-5-oxohexyl) phthalate (5oxo-MEHP) in each urine sample. We detected positive association between MnBP, MiBP, mono-2-ethylhexyl phthalate (MEHP), 5OH-MEHP, 5oxo-MEHP, 5cx-MECPP, mono-isononyl phthalate (MiNP), the sum of low (∑LMWP) and high molecular-weight phthalates (∑HMWP). and Tiffeneau–Pinelli index (the ratio of forced expiratory volume in 1/ forced vital capacity; FEV_1_/FVC; *p* = 0.001−0.04) and the percent predicted value (%PV) of FEV_1_/FVC (*p* = 0.005−0.05) and negative association between MiNP and peak expiratory flow (PEF; r = −0.31; *p* = 0.084). We observed a positive association between phthalate metabolites (MnBP, 5OH-MEHP, 5oxo-MEHP, 5cx-MECPP, 2cx-MMHP, ∑LMWP, and ∑HMWP) and waist-to-hip ratio (WHR; *p* = 0.003−0.09) and body shape index (ABSI; *p* = 0.039−0.09) and a negative association between MnBP, ∑LMWP, and hip circumference (*p* = 0.005−0.02). We detected association between concentrations of 5OH-MEHP, 5cx-MECPP, 5oxo-MEHP, and MnBP and consumption of food heating in plastic material in microwave (*p* = 0.02−0.04) and between probands who ate margarines and vegetable fat packed in plastic containers and concentration of MMP (*p* = 0.03). Results of multivariate regression indicated that exposure to phthalates could be linked with changing body structure, which subsequently affects values of pulmonary functions in firefighters.

## 1. Introduction

One of the worst fire seasons in Australia’s history showed the need to increase the number of firefighters to confront danger and to protect communities. Volunteer firefighting forces overwhelmingly represents an essential part of these efforts. In Slovakia, up until November of 2019, there were approximately 81,333 volunteer firefighters [1] and 3145 career firefighters [2]. Because of health risks, there is a strong need to protect and monitor the health of these emergency services.

Studies suggest that working conditions and hazards in this occupation vary according to different situations (e.g., wildland firefighter vs. municipal firefighters). On the other hand, all firefighters share some common working conditions. Serious chemical and physical hazards, primarily trauma, thermal injury, and smoke inhalation are obvious in occupational exposure of firefighters. This occupation has been studied intensively for evidence of chronic health effects. [3,4,5,6,7]. The most important of harmful ingredients in the firefighters’ working environment is fire smoke [8], and for the acute impact which occupational exposure factors have on the respiratory tract of firefighters, inhalation injury is mentioned in first place [9]. In the past, there were recognized toxic substances released during a fire which had been associated with adverse effects on the human body, such as carbon monoxide, carbon dioxide, hydrogen chloride, phosgene, chlorine, halogen acids, and many others [8]. Plastic production, despite all of the economic problems and environmental discussions, is growing worldwide at a rate of about 5 % per year. In atmospheric samples where plastics and refuse were burned in open fires detected among others as major components were even-carbon-chain n-alkanes; the plasticizer di-2-ethylhexyl phthalate; and the antioxidants and lubricants/antiadhesives Irganox 1076, Irgafos 168, and its oxidation product tris(2,4-di-tertbutylphenyl) phosphate. Major compounds in smoke from burning plastics include the non-source-specific n-alkanes (mainly even predominance), terephthalic acid, phthalates, and 4-hydroxybenzoic acid, with minor amounts of polycyclic aromatic hydrocarbons (including triphenylbenzenes) and tris(2,4-di-tert-butylphenyl)phosphate [10]. Medical research suggests the relationship between occupational exposure and its effects on health outcomes of firefighters, especially high temperatures; irritating, suffocating, and toxic gases; vapours; dust; and extinguishing powder [11]. Chronic, long-time exposure to the toxic substances may lead to the development of nonspecific bronchial hyperreactivity; chronic obstructive pulmonary disease (COPD); asthma; occupational asthma or lung cancer [9,12]; heat stress; musculoskeletal injuries [13]; risk of multiple myeloma; non-Hodgkin lymphoma; cancer of reproductive organs (prostate and testicular); increased risk for brain, stomach, colon, and rectum health problems among firefighters [14]; and risk of death from coronary heart disease [15]. Firefighters exposed to burning polyvinyl chloride (PVC) had more frequent and severe self-reported respiratory symptoms [16] and high prevalence rates of overweight and obesity [4]. Female firefighters reported that, nearly a quarter of their first pregnancies ended in miscarriage; rates of preterm delivery also were high [4]. Asbestos exposure in this type of occupation was associated with excess malignant mesothelioma [7].

Work duties and personal protective clothing may be responsible for chemical exposure of firefighters, e.g., to phthalates. Phthalates, used as plasticizers in polyvinyl plastic materials (wire sheathing, flooring, wall coverings, furnishings, and vinyl siding), cosmetic, and other daily products [17,18] were also linked to human health outcomes in various occupational environment [19,20,21,22] and were connected to change of temperatures in the workplace [23]. Phthalate diesters make up a class of chemicals identified as a frequent contaminant of firefighter gear [24]. The study of Stevenson et al. [25] reported that firefighters are exposed to high levels of di(2-ethylhexyl) phthalate (DEHP) that come from protective clothing, suggesting that firefighters are exposed to both estrogenic and antiestrogenic agents, possibly phthalates that may lead to health risks observed in this occupation as a result of perturbation of hormone homeostasis. Personal exposure of workers to air pollutants can be evaluated using biological monitoring techniques. Chemical analysis of environmental pollutants and associated metabolites in urine has been a common biological monitoring approach [26]. Also, occupational exposure can use this model of biomonitoring. The main objective of this exploratory study, therefore, was to evaluate exposure to phthalates from urine and analyses of health risk and occupational health problems of firefighters.

## 2. Materials and Methods

Studies were carried in two Slovakian Fire and Rescue Service Stations, studied as a pooled cohort of firefighters—males aged 24–53 years (*n* = 32), residing in districts of Central Slovakia. The study received approval from the Institutional Review Board of the Slovak Medical University. All participants gave written informed consent before any protocol-specific procedure was performed. There was a possibility to withdraw participation anytime during the study. Subjects diagnosed with respiratory illness or metabolic disorders with an incomplete questionnaire and those with prescription of bronchodilators or steroids were excluded.

### 2.1. Anthropometry and Spirometry

All measurements were conducted by the GPs (“gold standard practice”), performed by trained technicians. Body composition (weight, body fat percentage, muscle mass percentage, and visceral fat level) was estimated by The Omron BF510 (Kyoto, Japan). Body-mass index (BMI), waist-to-height ratio (WHtR), waist-to-hip ratio (WHR), fat mass indices (FMI), and fat-free mass indices (FFMI) were calculated. Body shape index (ABSI) was estimated by the following formula: ABSI = WC (waist circumference)/(BMI (2/3) × height (1/2)) [27]. Three manoeuvres according to European Respiratory Society/American Thoracic Society recommendations [28] were performed by spirometer Spirolab II (MIR, Rome, Italy) and Winspiro PRO software to tested forced expiratory volume in 1 s (FEV1, L), forced vital capacity (FVC, L), the ratio of FEV1 to FVC (FEV1/FVC, %), peak expiratory flow (PEF), vital capacity (VC, L), and maximal voluntary ventilation (MVV, L). The percent predicted value (% of PV) was calculated as the percentage of the participant measured value to the predicted value of that participant for each pulmonary parameter. Pulmonary Function Tests (PFT) were conducted by a trained technician.

### 2.2. Analyses of Phthalate

Firefighters provided first spot urine samples (2 × 2 mL) at the beginning of work, not earlier than 6:00 am, at the end of the workweek (Friday, work duration of at least 8 h per shift), which gives information about individual exposures during the last 24 h. Samples were stored in a transport box at 2–6 °C and then in the deep freezer at −73 °C until analysis. We used high-performance liquid chromatography (HPLC) and tandem mass spectrometry (MS/MS) (Infinity 1260 and 6410 triplequad, Agilent, Santa Clara, CA, USA) to quantified urinary concentration of compounds: mono-methyl phthalate (MMP), mono-ethyl phthalate (MEP), mono-isobutyl phthalate (MiBP), mono-n-butyl phthalate (MnBP), mono-benzyl phthalate (MBzP), mono-2-ethylhexyl phthalate (MEHP), mono(2-ethyl-5-hydroxyhexyl) phthalate (5OH-MEHP), mono(2-ethyl-5-oxohexyl) phthalate (5oxo-MEHP), mono(2-ethyl-5-carboxypentyl) phthalate (5cx-MECPP), mono(2-carboxymethyl-hexyl) phthalate (2cx-MMHP), and mono-isononyl phthalate (MiNP) by the method of Pilka et al. [23]. The method was certificated for occupational/environmental medical—toxicological analysis for the environmental medical field by EQUAS (External Quality Assessment Scheme) in the German External Quality Assessment Scheme.

The analytical standards were purchased from Cambridge isotope laboratories (Tewksbury, MA, USA). Briefly, 1 mL of urine was thawed, buffered with ammonium acetate, spiked with isotope-labeled phthalate standards β-glucuronidase enzyme (K12 E. Coli, Roche, Mannheim, Germany), and incubated at 37 °C. After deconjugation, samples were diluted with phosphate buffer (NaH_2_PO_4_ in H_3_PO_4_) and loaded on Solid-Phase Extraction (SPE) cartridges (ABS Elut Nexus, Agilent, Santa Clara, CA, USA). Cartridges were conditioned with acetonitrile followed by phosphate buffer before extraction. To remove the hydrophilic compound, SPE cartridges were flushed by formic acid (Merck KGaA, Darmstadt, Germany) and HPLC-grade water. Elution of analytes was performed by acetonitrile (Merck KGaA, Darmstadt, Germany) and ethylacetate (Sigma-Aldrich, Steinheim, Germany). Eluate was dried by nitrogen gas and reconstituted with 200 μL of H_2_O and acetonitrile (1:1). For HPLC, an Agilent Infinity 1260 liquid chromatography equipped with ZORBAX Eclipse phenyl-hexyl column (2.1 × 150 mm, 3 µm) was used. The separation was done using a nonlinear gradient program of mobile phases C_2_H_3_N (acetonitrile) and 0.1 % acetic acid (Fisher Scientific, Loughborough, UK) in H_2_O. For mass specific detection of phthalate metabolites, we used triplequad with electro-spray ionization (Agilent 6410). Chromatographic and mass spectrometric parameters are described in Table 1.

### 2.3. Statistics

Association between anthropometric parameters (BMI, FFMI, WHR, and WHtR) and pulmonary functions and concentrations of phthalate metabolites were examined by Pearson/Spearman correlation analysis. The nonparametric Mann–Whitney U (Wilcoxon rank-sum) test was used for analyses between the pack-year index, sports activity, and dynamic pulmonary function. For a description of urinary phthalate metabolites levels, means with standard deviations (SDs), medians, and the 5th and 95th percentiles of concentrations were computed. Multivariate regression analysis was used to estimate associations between pulmonary functions and anthropometric parameters adjusted to occupational exposure to phthalates, in which urinary phthalate concentrations were base 10 log-transformed. All statistical analyses were performed using the SPSS for Windows statistical package (version 14.0; SPSS Inc., Chicago, IL, USA). A difference was considered to be statistically significant when the *p*-value was ≤0.05 or ≤0.01.

## 3. Results

This is the first study from Slovakia on firefighters and their occupational environment to identify possible exposure to phthalates with possible health-related effects.

Self-related health condition seems to be good: only 15.63 % of probands (*n* = 5) reported pneumonia, and only 6.25 % (*n* = 2) indicated a more frequent occurrence of bronchitis in the past. One proband was treated for high blood pressure, and one was treated for allergy. Based on the questionnaire, morning cough was reported by 32.0 % of probands, cough during the day or at night was 15.0 %, and morning expectoration was almost 28.0 % of probands. Shortness of breath and wheezing were documented through the questionnaire in 38.0 %, and night attack breathlessness was in 3.5 % of probands. In PFT, probands reached sufficient results and scored above 80 % of the percentage distribution of functional parameters. The exception is the parameter MVV, where 1 proband (3.13 %) had mild deviation which means that it reached only 70 %–79 % from predicted values. A typical week physical activity survey was designed to identify the time and frequency spent by various physical activities during a typical week in the past month. Physical activities of firefighters are pretty good; probands on average engaged in physical activity for 56 min/day and 2.6 days/week. Running (25 %), hiking (21.9 %), ice hockey (18.7 %), and fire-fighting sport (15.6 %) belong to the most common types of physical activities among firefighters.

Baseline characteristic of anthropometric and pulmonary function parameters are described in Table 2. Despite the physical activity, only 9.4 % of examined career and volunteer firefighters reached normal weight, and on the other hand, high prevalence rates of overweight (BMI ≥ 25–29.9 kg/m; 68.7 %) and obesity (BMI ≥ 30kg/m; 21.9 %) were found. There was no statistically significant difference between the BMI values and the age categories and frequency of the physical activity. Based on WHR values of probands, the overweight criterion met 28.1 % and obese was 9.4 %. After considering WHtR, overweight was estimated at 50 % and obese was in 6.3 % of probands. Analyses of body composition revealed high values of FMI in 46.9 % of probands and very high values in 25 % of firefighters. On the other hand, analyses of FFMI, which reflected muscular components of the body, showed that 84.4 % of probands scored a high level of muscle fraction.

Analysis of urine samples detected the presence of phthalate metabolites MiBP, MnBP, 5OH-MEHP, 5oxo-MEHP, and 5cx- MECPP in each urine sample, followed by the presence of phthalate metabolites MEP, MEHP, and 2cx-MMHP in 93.75 % of samples; of MBzP in 78.14 % of samples; of MiNP in 21.87 % of samples; and of MMP in 12.5 % of samples. Descriptive statistics of urinary phthalate metabolites is shown in Table 3.

Although probands from urban areas reached a higher score of BMI, FFMI, WHtR, FVC, FEV_1_, VC, MVV, MEP, and MEHP, a MiBP in comparison to those with rural areas and, on the other hand, firefighters from rural areas scored higher FMI; WHR; PEF; FEV_1_/FVC; and concentrations of metabolites MnBP, MBzP, 5OH-MEHP, 5cx-MECPP, 5oxo-MEHP, and 2cx-MMHP, we did not observe any statistically significant differences according to type of residence of probands. Also, we did not observe statistical differences between the time passed since the last fire intervention (less/more than two days) and the concentration of phthalates. Correlation analyses between anthropometry parameters and pulmonary functions detected some positive but also negative associations highlighted in Table 4.

However, smoking as a modifying factor of respiratory function did not affect statistically significantly any of the monitored functions; we paradoxically observed higher values of Tiffeneau–Pinelli index and VC in active smokers. Median values of pulmonary functions according to smoking habits are shown in Table 5.

In the next step, we analyzed exposure to phthalates according to smoking habits. Smokers reached the highest median values of MEHP, 5OH-MEHP, 5oxo-MEHP, 5cx-MECPP, and MnBP in comparison to ex-smokers and nonsmokers. On the other hand, nonsmokers were more exposed to MEP, MBzP, and 2cx-MMHP than others (Table 6).

In the cohort, we found a positive association between phthalate metabolites (MiBP, MnBP, MEHP,5OH-MEHP, 5oxo-MEHP, 5cx-MECPP, MiNP, ∑LMWP, and ∑HMWP) and pulmonary functions FEV_1_/FVC and FEV_1_/FVC% of PV (Table 7). From phthalate metabolites, we detected negative association only between MiNP and PEF but only on the border of statistical significance (r = −0.31; *p* = 0.084).

Based on Spearman correlation analyses, we observed positive association between phthalate metabolites (MnBP, 5OH-MEHP, 5oxo-MEHP, 5cx-MECPP, 2cx-MMHP, ∑LMWP, and ∑HMWP) and anthropometry parameters (WHR and ABSI). Metabolite MiBP positively affected only the ABSI parameter. ABSI shed light on elucidating the predictive ability of abdominal obesity that cannot be attributed to BMI alone and is a predictor of total and cause-specific mortality. On the other hand, metabolites MiNP, MnBP, and ∑LMWP negatively affected hip circumference (Table 7).

Next, we examined using multivariate regression analysis how exposure to phthalates may affect the relationship between anthropometric parameters and the PFTs in firefighters. We found a relation on the border of statistical significance between the FEV_1_% of PV and anthropometric parameters FFMI (β = 3.343, *p* = 0.06) and WHtR (β = −87.552, *p* = 0.072); however, significant association appeared after adjustment to phthalates MMP (β = 3.543, *p* = 0.058) and MBzP (β = 3.77, *p* = 0.033; β = −96.209, *p* = 0.046, respectively).

We also observed statistical significant associations between FEV_1_% of PV and ABSI (β = −1040.1, *p* = 0.038), with increasing of significant effect after adjustment to phthalate 5cx-MECPP (β = −1128.0, *p* = 0.036), MEP (β = −1083.2, *p* = 0.036), 5OH-MEHP (β = −1139.5, *p* = 0.035), MiBP (β = −1163.5, *p* = 0.035), ∑HMWP (β = −1157.9, *p* = 0.033), ∑LMWP (β = −1181.7, *p* = 0.032), 5oxo-MEHP (β = −1149.3, *p* = 0.032), MEHP (β = −1168.2, *p* = 0.026), 2cx-MMHP (β = −1155.6, *p* = 0.03), and MBzP (β = −1155.8, *p* = 0.02).

The FEV_1_/FVC% of PV was related to anthropometric parameter WHtR but on the border of statistically significance (β = 56.164, *p* = 0.061); however, after adjustment to MMP, a significant association between this pulmonary function and WHtR appeared (β = 66.959, *p* = 0.023). Associations between pulmonary function FEV_1_/FVC% of PV and ABSI (β = 874.65, *p* = 0.03), WHR (β = 47.788, *p* = 0.051), and waist circumference (β = 0.363, *p* = 0.045) have been increased after adjustment to MMP (β = 875.98, *p* = 0.02; β = 56.031, *p* = 0.019; β = 0.466, *p* = 0.009). MiNP also acts as a cofounder which increased the association between FEV_1_/FVC% of PV and waist circumference (β = 0.399, *p* = 0.023).

We also detected a positive association between FVC% of PV, and FFMI (β = 4.597, *p* = 0.01) increased after adjustment to MBzP (β = 4.87, *p* = 0.007. The FVC% of PV was statistically significant related to anthropometric parameters WHR (β = −100.65, *p* = 0.012) and WHtR (β = −133.86, *p* = 0.05) and additionally has been increased after adjustment to MMP (β = −110.76, *p* = 0.006; β = −147.7, *p* = 0.002), MEP (β = −103.96, *p* = 0.011; β = −145.94, *p* = 0.004), and MBzP (β = −106.79, *p* = 0.009; β = −139.29, *p* = 0.004). The %PV of FVC was statistically significantly related to ABSI (β = −1819.02, *p* ≤ 0.001).

We also observed negative relation between MVV% of PV and waist circumference (β = −0.848, *p* = 0.034, with increasing significant effect after adjustment to phthalates MEP (β = −0.877, *p* = 0.032) and MiNP (β = −0.901, *p* = 0.025)); abdominal circumference (β = −0.931, *p* = 0.023 adjusted to MEP (β = −0.931, *p* = 0.023) and MiNP (β = −1.003, *p* = 0.013)); WHR (β = −117.46, *p* = 0.029 adjusted to MEP (β = −122.7, *p* = 0.026), MBzP (β = −123.51, *p* = 0.025), and ∑LMWP (β = −143.6, *p* = 0.017)); and WHtR (β = −199.19, *p* = 0.002 adjusted to MEP (β = −219.5), MBzP (β = −205.12), MiBP (β = −222.2), and ∑LMWP (β = −2323.7, *p* ≤ 0.001)). We also detected relation between MVV% of PV and ABSI (β = −1989.6, *p* = 0.002) with increasing significant effect after adjustment to ∑LMWP (β = −2323.7, *p* ≤ 0.001).

We observed positive association between pulmonary function VC% of PV and FFMI (β = 4.295, *p* = 0.023) increasing after adjustment to MEHP (β = 4.362, *p* = 0.022), MBzP (β = 4.466, *p* = 0.021), MnBP (β = 4.362, *p* = 0.02), ∑LMWP (β = 4.558, *p* = 0.017), and MiBP (β = 4.607, *p* = 0.016).

**Figure 1:**
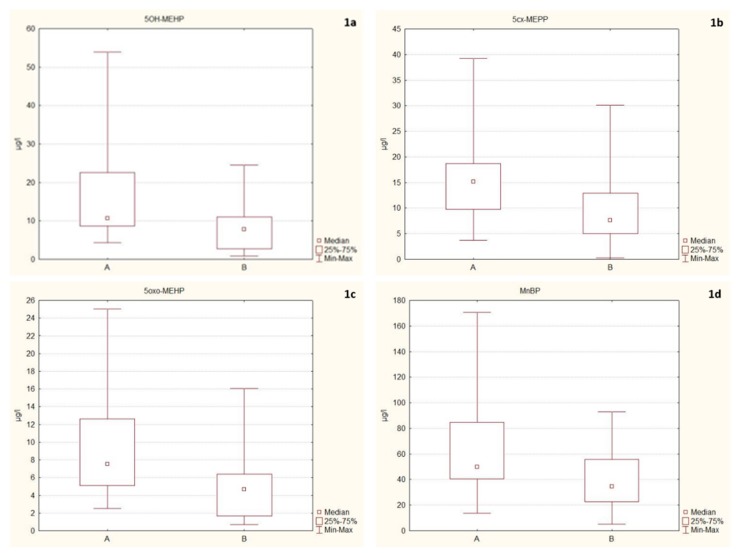
(**a**–**d**) Box and whisker plots of urinary concentrations of phthalate metabolites: **A**—Consumption of food heated in a plastic container; **B**—Nonconsumption of food heated in a plastic container; 5OH-MEHP, mono(2-ethyl-5-hydroxyhexyl) phthalate, *p* = 0.04; 5cx-MECPP, mono(2-ethyl-5-carboxypentyl) phthalate, *p* = 0.03; 5oxo-MEHP, mono(2-ethyl-5-oxohexyl) phthalate, *p* = 0.02; MnBP, Mono-n-butyl phthalate, *p* = 0.03.

In the next step, we assessed the effect of eating habits and lifestyle on phthalate exposure of firefighters. We observed statistical significantly higher concentrations of phthalates in probands who consumed food heated in a plastic container or wrapper during the previous 24 h before sample collection (A) in comparison to those who did not eat it (B), namely 5OH-MEHP (A, 106,644 μg/L vs. B, 78,040 μg/L; U = 67.00; *p* = 0.04); 5cx-MECPP (A, 151,403 μg/L vs. B, 75,916 μg/L; U = 63.00; *p* = 0.03); 5oxo-MEHP (A, 75,286 μg/L vs. B, 46,627 μg/L; U = 62.00; *p* = 0.02); and MnBP (A, 498,803 μg/L vs. B, 346,434 μg/L; U = 63.00; *p* = 0.03) (B; Figure 1a–d).

We detected significantly higher concentrations of MMP in probands who ate margarine and vegetable fat packed in a plastic container (median concentration A—17,023 μg/L) vs. those who did not eat it (B—56,462 μg/L; U = 17.5; *p* = 0.03).

We did not observe differences in probands according to their habits to drink liquid from plastic cups from the automatic machine in comparison to those who drink in another way. We also did not observe statistically significant differences according to the use of daily cosmetic products to use of PVC flooring material in household conditions and the use of personal protective equipment.

## 4. Discussion

In Slovakia, from all registered firefighters are 96.3 % volunteers and 3.7 % career firefighters, which is incomparable to data of US firefighters (volunteers 70 % vs. 30 % of career firefighters) [29]. This study was focused on the biomonitoring of occupational exposure to phthalates of firefighters and health-related outcomes. Except for the occupational environment, firefighters are potentially exposed to a wide range of xenobiotics contained in their daily lives, mainly through the environment, consumer products, food, and drinking water. Hence, it is possible to talk about overexposure or excessive exposure. For many chemicals, the health impacts associated with occupational exposure remain uncertain and overexposure may increase their health risks. Although the demands and hazards of firefighting have changed during the last decade, firefighters still routinely face risks. Therefore, biomonitoring of xenobiotics such as phthalates must be an essential form of protection.

In the study of Witt et al. [30], symptoms of firefighters from Poznan (Poland), such as morning cough (32 %), cough during the day or at night (21.3 %), morning expectoration (44.4 %), shortness of breath and wheezing (56.3 %), and night attacks of breathlessness (17.5 %) documented similar symptoms but in slightly higher frequency than ours. Studies suggests that chronic exposure to noxious agents in the workplace may occur in the development of nonspecific bronchial hyperreactivity, bronchiectasis, chronic obstructive pulmonary disease, asthma, occupational asthma, or lung cancer [9,31]. Although conflicting results exist, most experimental studies address the adjuvant effects of phthalates in immune responses [32]. A cohort study [16] reported that firefighters exposed to burning PVC at a warehouse fire had an increased risk of asthma-related respiratory symptoms. Bekö et al. [33] reported that di-(2-ethylhexyl) phthalate (DEHP) metabolites were associated with a higher risk of wheezing and bronchitis. In our study, only metabolite MiNP negatively affected PEF, which supports the study where phthalates have been considered as risk factors for asthma and allergic diseases [32,34,35]. On the other hand, paradoxically, our results pointed out positive associations between metabolites MiBP, MnBP, MEHP, 5OH-MEHP, 5oxo-MEHP, 5cx-MECPP, MiNP, ∑LMWP, and ∑HMWP and pulmonary parameter FEV_1_/FVC% of PV. Although these findings may seem surprising and are contrary to the hypothesis of phthalates as risk factors for asthma and allergic diseases, they also could play a role as harmful substances for pulmonary health. We could hypothesize on the bronchodilation effects of phthalates, but experimental studies are needed. Elucidating how phthalates induce asthma and allergic diseases was described in the study of Tsai et al. [32], where effects of phthalates in airway epithelial cells and the ensuing cell-cell interaction with bronchial smooth muscle cells, simulating the pathophysiology of airway remodelling in asthma and inflammatory airway diseases, were observed.

Results of pulmonary function test also suggest that, in our cohort, probands reach higher frequencies for FVC% of PV, FEV_1_% of PV, MVV% of PV, and Tiffaneau index about changes in the distribution of mild, moderate, and severe deviations than in a study from Poland [30]. It could be the effect of a relatively lower average age of our probands. In addition, in the mentioned study, the statistical relationship between the number of years of occupational exposure, the subjects’ age and smoking, and symptoms of respiratory disease were detected, which is contrary to our result.

In 2014, phthalate diesters as major contaminants of soiled firefighter gear have been studied [24,25]. Stevenson et al. [25] in their study also claim that new glove and hood extracts made from materials containing phthalates used by firefighters showed that significant estrogenic activity resulted in perturbation of hormone homeostasis. Phthalates contained in firefighters’ gear (either during production or during burning) are a worrying finding because phthalates are lipophilic, and the extreme heat helps phthalates enter through the skin and could modify their health. The presence of phthalate metabolites MiBP, MnBP, 5OH-MEHP, 5oxo-MEHP, and 5cx-MECPP in each urine sample can confirm this finding, although we did not observe the statistically significant association between phthalate exposure and the use of personal protective equipment. Further study is needed, and larger sample sizes may be needed to improve replicability. The effect of increasing temperature on the migration of phthalates also has been documented in some studies [23,36,37] although in the different environments and by increasing indoor and outdoor temperatures of the environments.

Some studies provided information about phthalate-contains in cigarettes [38,39] or higher concentration of phthalate metabolites in smokers [40,41]. Our results indicate that smokers reached slightly higher but not significantly different values of DEHP metabolites (MEHP, 5OH-MEHP, 5oxo-MEHP, and 5cx-MECPP) and MnBP, which support those studies, but on the other hand a lower concentration of MEP, MBzP, and 2cx-MMHP in comparison to ex-smokers and nonsmokers.

In comparison with our previous occupational studies, we can state that firefighters reached the lower median and mean concentration than hairdressing apprentices and communal services workers for MEP, MnBP, and MEHP [19,20,21], and lower concentrations of 5OH-MEHP, 5oxo-MEHP [20,21], MiNP [18], and MiBP [20]. Based on the conclusions of our studies mentioned above, we hypothesize that prolonged exposure (months to years) to low concentrations of phthalates may have a modifying effect on body composition, which can result in changes in pulmonary function. The effects on the health of prolonged but low-level exposure to phthalates may support the hypothesis about non-monotonic effect of phthalates. Some animal studies support this theory [42,43], but the potential non-monotonic effect of phthalates is still unclear and further study is needed.

High prevalence rates of overweight and obesity in our cohorts estimated by BMI are in accordance with study from USA [4]. Overweight in our study also confirms that 37.5 % of probands reached clinical criteria for overweight based on WHR and that 56.3 % probands reached overweight based on WHtR. It is necessary to state that these indices inform only about the distribution of fat in specific anatomical areas and do not fully reflect the proportion of muscle and fat components. On the other hand, the average value of fat content (FMI = 6.9) and muscle fraction (FFMI = 20.96) in the overall body composition indicates a high proportion of both monitored components.

Several chemicals in pliable plastics that can leach into food and concentration of phthalates from plastic containers increases with continued use of the containers [44]. Food ingestion is an important source of phthalate exposure [17,45]. We observed the statistically significantly higher concentrations of MnBP, 5OH-MEHP, 5oxo-MEHP, and 5cx-MECPP in probands who consumed food heated in a plastic container or wrapper and concentration of MMP in probands who ate margarine and vegetable fat packed in a plastic container 48 h before urine sampling. To ensure the safety of food contact materials, EU law provides binding rules that business operators must comply with, but contamination of food to these chemicals can come from contact with many materials and articles during its production, processing, storage, preparation, and serving before its eventual consumption. This contamination does not occur by the food manufacturer or the food container manufacturer but by the consumers itself. In our research, we noticed that firefighters carry food in their own containers (often not intended for transporting and heating food) or heat them in these containers in a microwave oven. We suppose that this pattern of behaviour could be the cause of phthalate exposure through ingestion.

In the study of Stevenson et al. [25], the authors state that firefighter’s gloves and hoods are impregnated with potent endocrine-disrupting agents and with estrogenic and antiestrogenic activity. In the context of some associations between the concentrations of phthalate metabolites and anthropometric parameters findings in our study, we hypothesize on the potential obesogenic effect of phthalates, potentially involved in weight gain by altering lipid homeostasis and by promoting adipogenesis and lipid accumulation. Effect of phthalates as potential obesogens was studied by Hao et al. [46,47], where di-(2-ethylhexyl) phthalate (DEHP) induced the expression of transcriptional factors peroxisome proliferator-activated receptor (PPAR) gamma, CCAAT/enhancer-binding protein (C/EBP) alpha and sterol regulatory element-binding factor 1 (Srebf1) as well as downstream target genes required for adipogenesis in vivo. Some studies also suggest that the associations between phthalates exposure and fat distribution and accumulation could be race/ethnicity- [48,49] and sex-specific [50]. Based on the results of multivariate regression between PFT and anthropometric parameters adjusted to phthalates and information mentioned above, we hypothesize that exposure to phthalates could change body composition and could affect values of pulmonary functions, particularly FEV_1_/ FVC, VC and PEF.

Limitations of our study are a small number of participants, the failure to adjust for potential confounders (other chemicals), and the failure to measure parent compounds, which may be subject to sample contamination. Non-monitored chemicals used in the workplace could play a role as co-exposure. Also, life habits and consumer practices of probands could affect concentrations of phthalates. The mixture effects of all pollutants representing occupational exposure have to be accounted for. The small sample size was one of the limitations of our study to realize those analyses. For this reason, our results cannot be generalized to the broader community based on this study alone

## 5. Conclusions

Even though firefighters are exposed to many occupational hazards, studies regarding these risks are still insufficient. Firefighters are an occupational group subject which should be examined annually by mandatory periodic inspections. Our results indicate that biomonitoring of phthalate exposure using spot urine can provide useful information concerning the workplace where personal air monitoring equipment is not available. Phthalates exposure and health-related effects appear to signal for studies to the elimination of risk in an occupational environment of firefighters. To reduce the potential risk of phthalate occupational exposure, we propose that firefighters should have a second set of turnout gear to wear in case there is a fire while the first set is being cleaned. Adequate attention should be given to education about potential hazards and preventive strategies in this occupational environment. Appropriate preventive strategy by pulmonary function test in periodical monitoring can detect early signs of pulmonary dysfunctions. It would be advisable to continue the extensive research on the working environment of firefighters and its impact on the living organism. Future research should be focused on the following:

- the usage and industrial hygiene practices related to the equipment, including cleaning patterns, length of use, and storage practices to prevent deposition of contaminants on firefighter protective equipment
- health effects related to potential absorption through the skin

## Figures and Tables

**Table 1:** Phthalate monoesters: chromatographic and mass spectrometric parameters.

Metabolite	Precursor Ion (m/z)	Product ion (m/z)	LOD (µg/L)
MMP	179	77	3.41
MMP labeled	183	79	
MEP	193.1	77	3.23
MEP labeled	197.1	79	
MiBP*	221.1	76.9	0.67
MnBP	221.1	76.9	0.67
MnBP labeled	225.1	78.9	
MBzP	255.1	76.9	0.19
MBzP labeled	259.1	79	
MEHP	277.1	133.9	0.34
MEHP labeled	281.1	137.1	
5OH-MEHP	293.1	121	1.8
5OH-MEHP labeled	297.2	124.1	
5cx-MECPP	307.1	159.1	0.23
5cx-MECPP labeled	311.1	159.1	
5oxo-MEHP	291.2	121	0.48
5oxo-MEHP labeled	295.1	124	
2cx-MMHP	307.2	159.2	0.21
2cx-MMHP labeled	311.2	159.1	
MiNP	291.2	141.2	0.57
MiNP labeled	295.3	79	

Notes: MMP, Mono-methyl phthalate; MEP, mono-ethyl phthalate. MiBP, mono-isobutyl phthalate; MnBP, Mono-n-butyl phthalate, MBzP, mono-benzyl phthalate; MEHP, Mono-2-ethylhexyl phthalate; 5OH-MEHP, mono(2-ethyl-5-hydroxyhexyl) phthalate; 5oxo-MEHP, mono(2-ethyl-5-oxohexyl) phthalate; 5cx-MECPP, mono(2-ethyl-5-carboxypentyl) phthalate; 2cx-MMHP, mono(2-carboxymethylhexyl) phthalate; MiNP, mono-isononyl phthalate; LOD, limit of detection. *Semiquantitative by MnBP.

**Table 2:** Baseline characteristic of anthropometric and pulmonary function parameters.

	Mean	Median	Minimum	Maximum	Percentile 95	SD
**Age (year)**	38.22	39.00	24.00	53.00	50.00	8.23
**Height (cm)**	180.28	179.50	165.00	191.00	191.00	6.43
**Weight (kg)**	90.72	89.00	72.00	114.00	111.00	9.98
**BMI**	27.87	27.91	23.30	32.74	31.25	2.23
**FMI**	6.90	6.94	4.35	10.72	10.14	1.80
**FFMI**	9.96	9.92	8.85	11.26	11.09	0.53
**Transversal Chest Diameter (cm)**	30.11	30.50	21.20	34.40	33.00	2.72
**Sagital Chest Diameter (cm)**	23.08	23.20	19.40	30.20	27.60	2.59
**Waist Circumference (cm)**	90.20	91.55	77.50	101.00	99.60	6.46
**Hip Circumference (cm)**	99.06	98.25	90.80	113.20	109.50	5.27
**Abdomen Circumference (cm)**	92.56	92.75	79.10	104.00	102.70	6.39
**WHR**	0.93	0.93	0.80	1.03	1.02	0.05
**WHtR**	0.50	0.51	0.42	0.58	0.57	0.04
**FVC% of PV**	104.01	103.82	80.80	122.96	121.73	10.95
**FEV**_**1**_ **% of PV**	103.31	103.44	82.37	125.65	121.87	10.66
**PEF% of PV**	92.77	91.65	72.69	112.61	108.64	11.34
**FEV**_**1**_ **/FVC**	81.84	82.85	67.20	89.70	89.10	4.74
**FEV**_**1**_ **/FVC% of PV**	101.93	102.57	82.14	114.72	109.22	6.57
**VC% of PV**	103.02	102.10	69.44	124.70	122.16	11.49
**MVV% of PV**	106.01	107.46	71.46	131.52	130.59	14.58

Notes: BMI, Body mass index; FMI, fat mass index; FFMI, fat-free mass index; WHR, waist-to-hip ratio; WHtR, waist-to-height ratio; FVC% of PV, % of predicted values of forced vital capacity; FEV_1_% of PV, % of predicted values of forced expiratory volume in 1 s; FEV_1_/FVC, ratio of forced expiratory volume in 1 s to forced vital capacity; PEF% of PV, % of predicted values of peak expiratory flow; FEV_1_/FVC% of PV, % of predicted values of ratio of forced expiratory volume in 1 s to forced vital capacity; VC% of PV, % of predicted values of vital capacity; MVV% of PV, % of predicted values of maximum voluntary ventilation.

**Table 3:** Descriptive statistic of urinary phthalate metabolite (μg/L).

					Percentile			
	%	Mean	SD	Min.	5.	95.	Max.	Median
MMP	12.50	3.02	5.22	1.70	1.70	9.60	30.71	1.70
MEP	93.75	64.55	222.55	1.26	1.28	261.58	1251.61	9.86
MiBP	100.00	29.36	19.69	75.68	3.70	66.12	75.68	23.95
MnBP	100.00	56.26	37.87	5.26	6.19	150.24	170.78	45.17
MBzP	78.14	0.22	0.27	0.00	0.02	0.64	1.39	0.12
MEHP	93.75	5.94	5.47	0.24	0.24	17.22	21.85	3.93
5OH-MEHP	100.00	13.33	11.21	0.81	1.28	33.27	53.99	9.82
5oxo-MEHP	100.00	7.86	5.75	0.69	0.87	18.72	25.02	6.03
5cx-MECPP	100.00	14.63	10.78	0.30	0.49	37.55	39.22	11.58
2cx-MMHP	93.75	3.61	2.84	0.15	0.15	10.20	11.21	2.86
MiNP	21.87	0.91	1.74	0.28	0.28	3.86	9.36	0.28

Notes: MMP, Mono-methyl phthalate; MEP, mono-ethyl phthalate; MiBP, mono-isobutyl phthalate; MnBP, Mono-n-butyl phthalate; MBzP, mono-benzyl phthalate; MEHP, Mono-2-ethylhexyl phthalate; 5OH-MEHP, mono(2-ethyl-5-hydroxyhexyl) phthalate); 5oxo-MEHP, mono(2-ethyl-5-oxohexyl) phthalate; 5cx-MECPP, mono(2-ethyl-5-carboxypentyl) phthalate; 2cx-MMHP, mono(2-carboxymethylhexyl) phthalate; MiNP, mono-isononyl phthalate.

**Table 4:** Bivariate analysis between Pulmonary Function Tests (PFT) parameters and anthropometric parameters in firefighters (*n* = 32), Pearson correlation coefficient (*p* value).

	Pulmonary Function						
Anthropometric Parameters	%FEV_1_ of PV	FEV_1_/FVC	%FEV_1_/FVC of PV	VC	%VC of PV	MVV	%MVV of PV
**Waist circumference**			0.357 (0.045)			−0.428 (0.014)	−0.376 (0.034)
**Hip circumference**							
**Abdominal circumference**						−0.4060 (0.021)	−0.397 (0.025)
**ABSI**	−0.369 (0.038)	0.384 (0.003)	0.503 (0.003)	−0.409 (0.020)		−0.614 (0.000)	
**WHR**	−0.322 (0.072)		0.348 (0.051)			−0.499 (0.004)	−0.386 (0.029)
**WHtR**			0.335 (0.061)	−0.351 (0.049)		−0.677 (0.000)	−0.536 (0.002)
**FMI**						−0.351 (0.049)	−0.536 (0.002)
**FFMI**	0.336 (0.06)			0.371 (0.036)	0.401 (0.023)		

Notes: PFT, Pulmonary Function Test; FEV_1_ of PV, % of predicted values of forced expiratory volume in 1 s (L); FEV_1_/FVC, the ratio of FEV_1_ to FVC (%); VC, vital capacity (L); %VC of PV, % of predicted values of vital capacity; %MVV, maximal voluntary ventilation (L); %MVV of PV, % of predicted values of maximum voluntary ventilation; ABSI, A Body Shape Index; WHtR, waist-to-height ratio; WHR, waist-to-hip ratio; FFMI, fat-free mass index; FMI, fat mass index.

**Table 5:** Pulmonary function test (median ± SD) in the studied group affected by smoking habits.

Parameter	Smokers	Ex-smokers	No-smokers
	*n* = 8	*n* = 6	*n* = 18
FVC% of PV	97.29 ± 10.66	105.48 ± 12.23	105.27 ± 9.91
FEV_1_% of PV	102.16 ± 9.42	99.46 ± 12.18	104.51 ± 9.70
FEV_1_/FVC	84.1 ± 4.14	81.15 ± 4.78	82.85 ± 5.12
PEF% of PV	84.84 ± 10.71	98.12 ± 11.04	91.65 ± 11.18
VC% of PV	104.14 ± 11.87	100.88 ± 15.12	102.10 ± 9.53
MVV% of PV	106.19 ± 16.18	100.31 ± 13.71	111.39 ± 13.71
p/y index	5.73 ± 10.86	5.56 ± 10.24	

Notes: FVC% of PV, % of predicted values of forced vital capacity; FEV_1_% of PV, % of predicted values of forced expiratory volume in 1 s; FEV_1_/FVC, ratio of forced expiratory volume in 1 s to forced vital capacity; PEF% of PV, % of predicted values of peak expiratory flow; VC% of PV, % of predicted values of vital capacity; MVV% of PV, % of predicted values of maximum voluntary ventilation; p/y index, pack-year index.

**Table 6:** Phthalate metabolites (µg/L) in the studied group divided by smoking habits.

Parameter	Smokers			Ex-smokers			No-smokers		
	*n* = 8			*n* = 6			*n* = 18		
	Mean	Median	SD	Mean	Median	SD	Mean	Median	SD
MMP	1.7023	1.7023	0.0000	1.7023	1.7023		4.0407	1.7023	6.8645
MEP	5.8486	4.7282	4.1478	33.689	12.615	57.470	100.93	17.147	293.22
MiBP	23.469	20.647	13.9344	29.443	27.813	19.609	31.9565	0.7031	22.146
MnBP	66.202	58.781	44.795	43.106	38.465	31.631	56.234	45.172	37.236
MBzP	0.1222	0.1289	0.0841	0.1313	0.0942	0.1072	0.2905	0.1554	0.3324
MEHP	6.0543	5.5221	4.6057	2.8422	2.2027	3.0789	6.9261	4.7507	6.2114
5OH-MEHP	10.945	9.8189	6.0323	10.941	7.4785	10.848	15.189	9.5144	13.078
5oxo-MEHP	7.2288	6.5570	4.0405	5.7417	4.1954	4.9495	8.8499	5.7713	6.5920
5cx-MECPP	12.973	12.011	8.4821	13.585	10.172	12.861	15.717	10.564	11.434
2cx-MMHP	3.1220	2.8272	2.1284	2.8610	2.4416	2.4689	4.0833	3.1828	4.2449
MiNP	1.8644	0.2825	3.2763	0.2825	0.2825		0.6922	0.2825	0.7073

Notes: MMP, Mono-methyl phthalate; MEP, mono-ethyl phthalate; MiBP, mono-isobutyl phthalate; MnBP, Mono-n-butyl phthalate; MBzP, mono-benzyl phthalate; MEHP, Mono-2-ethylhexyl phthalate; 5OH-MEHP, mono(2-ethyl-5-hydroxyhexyl) phthalate); 5oxo-MEHP, mono(2-ethyl-5-oxohexyl) phthalate; 5cx-MECPP, mono(2-ethyl-5-carboxypentyl) phthalate; 2cx-MMHP, mono(2-carboxymethylhexyl) phthalate; MiNP, mono-isononyl phthalate; LOD, limit of detection.

**Table 7:** Bivariate analysis between phthalate metabolites and PFT and anthropometry in firefighters (*n* = 32), Spearman correlation coefficient (*p* value).

	Pulmonary Function			Anthropometry Parameters		
Phthalate Metabolites	FEV_1_/FVC	%PV of FEV_1_/FVC	PEF	Hip Circumference	WHR	ABSI
MiBP	0.365(0.040)	0.379(0.032)				0.316(0.078)
MnBP	0.376(0.034)	0.383 (0.030)		−0.484(0.005)	0.511(0.003)	0.302(0.093)
MEHP	0.542(0.001)	0.482(0.005)				
5OH-MEHP	0.529(0.002)	0.456(0.009)			0.433(0.013)	0.367(0.039)
5oxo-MEHP	0.549(0.001)	0.480(0.005)			0.436(0.013)	0.338(0.059)
5cx-MECPP	0.544(0.001)	0.459 (0.008)			0.357(0.045)	0.310(0.084)
2cx-MMHP	0.402(0.023)	0.342 (0.055)			0.300(0.096)	0.322(0.072)
MiNP	0.310(0.075)	0.319 (0.075)	−0.310(0.084)	−0.308(0.086)		
∑LMWP	0.417(0.017)	0.423(0.016)		−0.404(0.022)	0.395(0.025)	0.353(0.047)
∑HMWP	0.533(0.002)	0.463(0.007)			0.385(0.030)	0.335(0.061)

Notes: FEV_1_/FVC, the ratio of FEV_1_ to FVC (%); PEF, peak expiratory flow; MiBP, mono-isobutyl phthalate; MnBP, Mono-n-butyl phthalate; MEHP, Mono-2-ethylhexyl phthalate; 5OH-MEHP, mono(2-ethyl-5-hydroxyhexyl) phthalate; 5oxo-MEHP, mono(2-ethyl-5-oxohexyl) phthalate; 5cx-MECPP, mono(2-ethyl-5-carboxypentyl) phthalate; 2cx-MMHP, mono(2-carboxymethylhexyl) phthalate; MiNP, mono-isononyl phthalate.

## References

[1] Kolena B., Parčiš I.J. (2020). Personal Communication.

[2] Kolena B., Križanová n.M.K. (2020). Personal Communication.

[3] Guidotti T.L., Clough V.M. (1992). Occupational health concerns of firefighting. Annu. Rev. Publ. Health.

[4] Poston W.S.C., Haddock C.K., Jahnke S.A., Jitnarin N., Tuley B.C., Kales S.N. (2011). The prevalence of overweight, obesity, and substandard fitness in a population-based firefighter cohort. J. Occup. Environ. Med..

[5] Soteriades E.S., Smith D.L., Tsismenakis A.J., Baur D.M., Kales S.N. (2011). Cardiovascular disease in US firefighters: A systematic review. Cardiol. Rev..

[6] Soteriades E.S., Targino M.C., Talias M.A., Hauser R., Kawachi I., Christiani D.C., Kales S.N. (2011). Obesity and risk of LVH and ECG abnormalities in US firefighters. J. Occup. Environ. Med..

[7] Daniels R.D., Kubale T.L., Yiin J.H., Dahm M.M., Hales T.R., Baris D., Zahm S.H., Beaumont J.J., Waters K.M., Pinkerton L.E. (2014). Mortality and cancer incidence in a pooled cohort of US fire fighters from San Francisco, Chicago and Philadelphia (1950–2009). Occup. Environ. Med..

[8] Hartzell G.E., Packham S.C., Switzer W.G. (1983). Toxic products from fires. Am. Ind. Hyg. Assoc. J..

[9] Wright J.L., Cagle P., Churg A., Colby T.V., Myers J. (1992). Diseases of the small airways. Am. Rev. Respir. Dis..

[10] Simoneit B.R.T., Medeiros P.M., Didyk B.M. (2005). Combustion products of plastics as indicators for refuse burning in the atmosphere. Environ. Sci. Technol..

[11] Krzywiecki A., Płusa T. (1993). Uszkodzenie tkanki płucnej wywołane oparzeniem termiczny m. Stany Zagrożenia Życia w Pneumonologii i Alergologii.

[12] Horsfield K., Cooper F.M., Buckman M.P., Guyatt A.R., Cumming G. (1998). Respiratory symptoms in West Sussex Firemen. Br. J. Ind. Med..

[13] Shaikha A., Farah A., Israa A., Waleed A., Lulwa A., Abdulaziz A., Mariam S., Noura A., Abdulaziz A., Sundus B. (2017). Occupational hazards among firefighters in Kuwait 2016. Curr. Trends. Clin. Med. Imaging.

[14] LeMasters G.K., Genaidy A.M., Succop P., Deddens J., Sobeih T., Barriera-Viruet H., Dunning K., Lockey J. (2006). Cancer risk among firefighters: A review and meta-analysis of 32 studies. J. Occup. Environ. Med..

[15] Kales S.N., Soteriades E.S., Christophi C.A., Christiani D.C. (2007). Emergency duties and deaths from heart disease among firefighters in the United States. N. Engl. J. Med..

[16] Markowitz J.S. (1989). Self-reported short- and long-term respiratory effects among pvc-exposed firefighters. Arch. Environ. Health.

[17] Schettler T., Skakkebæk N.E., De Kretser D., Leffers H. (2006). Human exposure to phthalates via consumer products. Int. J. Androl..

[18] Heudorf U., Mersch-Sundermann V., Angerer J. (2007). Phthalates: Toxicology and exposure. Int. J. Hyg. Environ. Health.

[19] Kolena B., Petrovicova I., Pilka T., Pucherova Z., Munk M., Matula B., Vankova V., Petlus P., Jenisova Z., Rozova Z. (2014). Phthalate exposure and health-related outcomes in specific types of work environment. Int. J. Environ. Res. Public Health.

[20] Kolena B., Petrovicová I., Šidlovská M., Pilka T., Neuschlová M., Valentová I., Rybanský L., Trnovec T. (2017). Occupational phthalate exposure and health outcomes among hairdressing apprentices. Hum. Exp. Toxicol..

[21] Kolena B., Petrovicova I., Sidlovska M., Hlisnikova H., Tomasovova E., Zoldakova V., Trajtelova H., Rybansky L., Wimmerova S., Trnovec T. (2019). Phthalates exposure and occupational symptoms among Slovakian hairdressing apprentices. Appl. Sci..

[22] Petrovičová I., Kolena B., Šidlovská M., Pilka T., Wimmerová S., Trnovec T. (2016). Occupational exposure to phthalates in relation to gender, consumer practices and body composition. Environ. Sci. Pollut. Res..

[23] Pilka T., Petrovicova I., Kolena B., Zatko T., Trnovec T. (2014). Relationship between variation of seasonal temperature and extent of occupational exposure to phthalates. Environ. Sci. Pollut. Res..

[24] Fabian T.Z., Borgerson J.L., Gandhi P.D., Baxter C.S., Ross C.S., Lockey J.E., Dalton J.M. (2014). Characterization of firefighter smoke exposure. Fire Technol..

[25] Stevenson M., Alexander B., Stuart Baxter C., Leung Y.K. (2015). Evaluating endocrine disruption activity of deposits on firefighting gear using a sensitive and high throughput screening method. J. Occup. Environ. Med..

[26] Lauwerys R.R., Hoet P. (2001). Industrial Chemical Exposure: Guidelines for Biological Monitoring.

[27] Krakauer N.Y., Krakauer J.C. (2012). A new body shape index predicts mortality hazard independently of body mass index. PLoS ONE.

[28] Miller M.R., Hankinson J., Brusasco V., Burgos F., Casaburi R., Coates A., Crapo R., Enright P., van der Grinten C.P.M., Gustafsson P. (2005). Standardisation of spirometry. Eur. Respir. J..

[29] (2019). NFPA Report—U.S. Fire Department Profile. https://www.nfpa.org/News-and-Research/Data-research-and-tools/Emergency-Responders/US-fire-department-profile.

[30] Witt M., Goniewicz M., Pawłowski W., Goniewicz K., Biczysko W. (2017). Analysis of the impact of harmful factors in the workplace on functioning of the respiratory system of firefighters. Ann. Agric. Environ. Med..

[31] Hardy’ T.S., Weill H. (1995). Crystalline silica: Risks and policy. Environ. Health Perspect..

[32] Tsai M.J., Kuo P.L., Ko Y.C. (2012). The association between phthalate exposure and asthma. Kaohsiung J. Med Sci..

[33] Bekö G., Callesen M., Weschler C.J., Toftum J., Langer S., Sigsgaard T., Høst A., Kold Jensen T., Clausen G. (2015). Phthalate exposure through different pathways and allergic sensitization in preschool children with asthma, allergic rhinoconjunctivitis and atopic dermatitis. Environ. Res..

[34] Brunetti G., Moscato G. (1984). Bronchial asthma due to occupational exposure to a dioctylphthalate. Description of a case. La Med. Del Lav..

[35] Jaakkola J.J.K., Knight T.L. (2008). The role of exposure to phthalates from polyvinyl chloride products in the development of asthma and allergies: A systematic review and meta-analysis. Environ. Health Perspect..

[36] Fujii M., Shinohara N., Lim A., Otake T., Kumagai K., Yanagisawa Y. (2003). A study on emission of phthalate esters from plastic materials using a passive flux sampler. Atmos. Environ..

[37] Clausen P.A., Liu Z., Kofoed-Sørensen V., Little J., Wolkoff P. (2012). Influence of temperature on the emission of Di-(2-ethylhexyl)phthalate (DEHP) from PVC flooring in the emission cell FLEC. Environ. Sci. Technol..

[38] Hoffmann D., Hoffmann I. (1988). Chemistry and toxicology. Smok. Tobacco Control Monograpg..

[39] Talhout R., Schulz T., Florek E., van Benthem J., Wester P., Opperhuizen A. (2011). Hazardous compounds in tobacco smoke. Int. J. Environ. Res. Public Health.

[40] Duty S.M., Ackerman R.M., Calafat A.M., Hauser R. (2005). Personal care product use predicts urinary concentrations of some phthalate monoesters. Environ. Health Perspect..

[41] Geens T., Bruckers L., Covaci A., Schoeters G., Fierens T., Sioen I., Vanermen G., Baeyens W., Morrens B., Loots I. (2014). Determinants of bisphenol A and phthalate metabolites in urine of Flemish adolescents. Environ. Res..

[42] Do R.P., Stahlhut R.W., Ponzi D., vom Saal F.S., Taylor J.A. (2012). Non-monotonic dose effects of in utero exposure to di(2-ethylhexyl) phthalate (DEHP) on testicular and serum testosterone and anogenital distance in male mouse fetuses. Reprod. Toxicol..

[43] Santangeli S., Maradonna F., Zanardini M., Notarstefano V., Gioacchini G., Forner-Piquer I., Habibi H., Carnevali O. (2017). Effects of diisononyl phthalate on Danio rerio reproduction. Environ. Pollut..

[44] Moreira M.A., André L.C., Cardeal Z.L. (2013). Analysis of phthalate migration to food simulants in plastic containers during microwave operations. Int. J. Environ. Res. Public Health.

[45] Ji Y., Wang F., Zhang L., Shan C., Bai Z., Sun Z., Liu L., Shen B. (2014). A comprehensive assessment of human exposure to phthalates from environmental media and food in Tianjin, China. J. Hazard. Mater..

[46] Hao C., Cheng X., Xia H., Ma X. (2012). The endocrine disruptor mono-(2-ethylhexyl) phthalate promotes adipocyte differentiation and induces obesity in mice. Biosci. Rep..

[47] Hao C., Cheng X., Guo J., Xia H., Ma X. (2013). Perinatal exposure to diethyl-hexyl-phthalate induces obesity in mice. Front. Biosci. Elite.

[48] Trasande L., Spanier A.J., Sathyanarayana S., Attina T.M., Blustein J. (2013). Urinary phthalates and increased insulin resistance in adolescents. Pediatrics.

[49] Trasande L., Attina T.M., Sathyanarayana S., Spanier A.J., Blustein J. (2013). Race/ethnicity-specific associations of urinary phthalates with childhood body mass in a nationally representative sample. Environ. Health Perspect..

[50] Lind P.M., Roos V., Rönn M., Johansson L., Ahlström H., Kullberg J., Lind L. (2012). Serum concentrations of phthalate metabolites are related to abdominal fat distribution two years later in elderly women. Environ. Health Glob. Access Sci. Source.

